# Fluorescent Dendritic Micro-Hydrogels: Synthesis, Analysis and Use in Single-Cell Detection

**DOI:** 10.3390/molecules23040936

**Published:** 2018-04-18

**Authors:** Lisa Christadore, Mark W. Grinstaff, Scott E. Schaus

**Affiliations:** 1 Department of Chemistry, Boston University, Boston, MA 02215, USA; lmchristadore1@gmail.com; 2 Departments of Biomedical Engineering and Medicine, Boston University, Boston, MA 02215, USA

**Keywords:** dendrimer, hydrogel, screening, crosslinking, fluorescence

## Abstract

Hydrogels are of keen interest for a wide range of medical and biotechnological applications including as 3D substrate structures for the detection of proteins, nucleic acids, and cells. Hydrogel parameters such as polymer wt % and crosslink density are typically altered for a specific application; now, fluorescence can be incorporated into such criteria by specific macromonomer selection. Intrinsic fluorescence was observed at λ_max_ 445 nm from hydrogels polymerized from lysine and aldehyde- terminated poly(ethylene glycol) macromonomers upon excitation with visible light. The hydrogel’s photochemical properties are consistent with formation of a nitrone functionality. Printed hydrogels of 150 μm were used to detect individual cell adherence via a decreased in fluorescence. The use of such intrinsically fluorescent hydrogels as a platform for cell sorting and detection expands the current repertoire of tools available.

## 1. Introduction

Dendrimers are well-defined macromolecules with unique properties that are advantageous for a wide range of applications [1,2,3,4], with particular utility in medical and biotechnological systems [5,6,7,8,9,10]. For example, dendrimer-based delivery vehicles for anticancer drugs provide new approaches to tune cellular uptake and pharmacokinetics [11,12,13,14,15,16,17,18,19,20]. Multifunctionalized dendritic macromonomers are of interest for the formation of crosslinked hydrogels to repair bone [21] and soft tissues [22,23,24,25,26,27,28], to engineer cartilage tissue scaffolds [29,30], to deliver drugs [31,32], and to prepare microarrays for screening protein and nucleic acid interactions [33]. The latter study led us to the discovery of intrinsic fluorescence emitted by hydrogels prepared from PEG-(CHO)_2_ and PEG-(Lys)_2_, PEG-(Lys_2_)_2_, or [G1]-Lys-NH_2_ macromonomers. The macromonomers rapidly polymerized on aldehyde-coated slides to yield Schiff-base crosslinked hydrogels. When the hydrogel microarrays were analyzed with a fluorescent scanner, distinct fluorescence was observed that persisted over multiple days and washes with aqueous buffer. Herein, we describe the visible fluorescence, propose the origin of its emission, and develop a hydrogel label-free single-cell assay.

Previous studies described fluorescence from amine-containing poly(amido amine) and poly(propylene imine) dendrimers in solution with emission maximums between 440 and 450 nm upon excitation at ≈380 nm [34,35,36,37,38]. Several groups reported that dendrimer fluorescence intensified with prolonged exposure to oxygen. Furthermore, emission intensities increased linearly with dendrimer concentrations and were unaffected by terminal group composition. These results suggested that the fluorescence was directly related to the dendrimers’ common tertiary amine and/or amide backbones. Recent studies by Qian et al. suggest that the imine bond in the imidic acid group present within the PAMAM structure is responsible for emission [39,40]. In our studies, we propose that aerobic oxidation of the imine may be responsible for the fluorescence observed from the Schiff-base crosslinked hydrogels.

## 2. Results and Discussion

**Figure 1:**
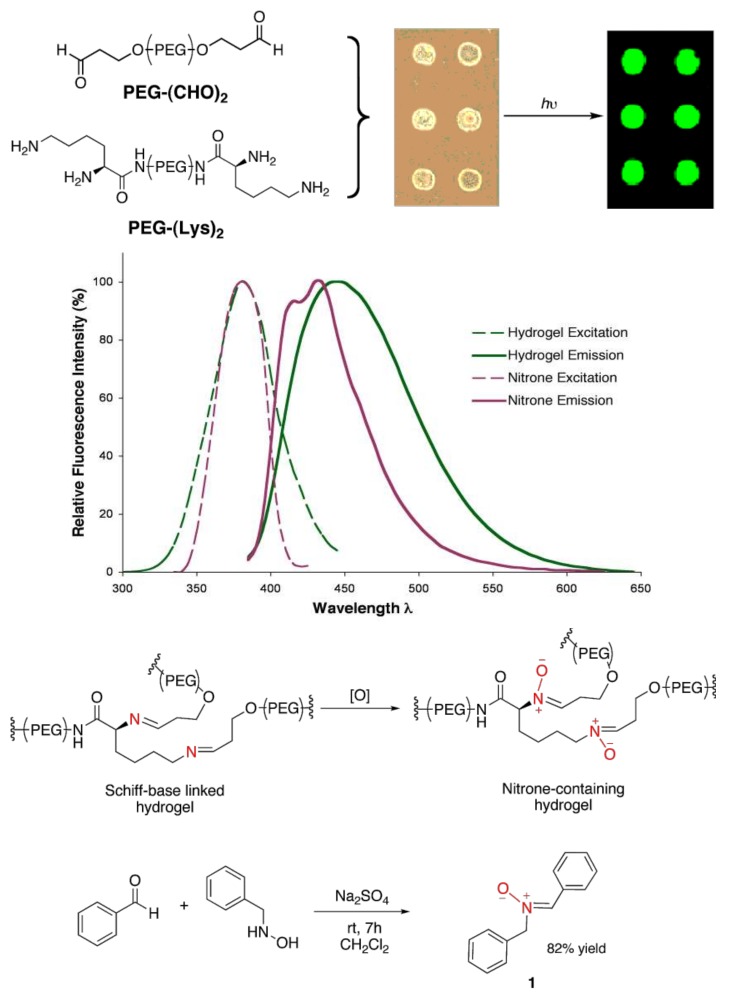
(**top**) PEG-(CHO)_2_ and PEG-(Lys)_2_ were mixed in situ and dispensed onto aldehyde-coated slides to form 150 μm hydrogel chambers using an OmniGrid accent microarrayer. Fluorescence emission was observed between 550–590 nm on a GenePix 4000B microarray scanner. (**middle**) Fluorescence spectra of the Schiff-base linked hydrogel, 14 wt % 2:1 PEG-(CHO)_2_:PEG-(Lys)_2_, polymerized in HEPES buffer pH 7.4 for 24 h (green), and (Z)-*N*-benzyl-α-phenylnitrone, **1**, in ethanol at 2.8 wt % immediately following solvation (purple) (**bottom**). Proposed air oxidation scheme of the hydrogel imine linkages to nitrones (**top**), and synthesis scheme of **1** (**bottom**).

We employed steady-state spectrofluorimetry and confocal fluorescence microscopy to analyze hydrogel fluorescence (Figure 1, top). Figure 1 middle depicts the excitation and emission fluorescence profiles of the Schiff-base crosslinked hydrogel. The excitation maximum was observed at 380 nm, and emission occurred over a broad range with a maximum at 445 nm. These results indicated a unique fluorescent structure existed within the hydrogel network that exhibited a relatively large Stokes shift (65 nm) and a wide emission bandwidth (FWHM = 89 nm). Fluorescence is not observed with amide-crosslinked hydrogels prepared from the reaction between Lys-terminated and PEG-(NHS)_2_ macromonomers.

We next addressed the possible chemical structure and mechanism responsible for the unique fluorescence properties of the hydrogel. Specifically, the imine functionality and the photochemical properties of its oxidation products were investigated. Previous studies have demonstrated nitrone formation by aerobic oxidation of secondary amines and imines. Such syntheses have utilized oxygen and hydrogen peroxide as primary oxidants, typically in conjunction with transition metal [41,42,43,44,45] and flavin-derived [46,47] catalysts. Thus, we proposed that air oxidation of the imine to the nitrone may be responsible for the observed hydrogel fluorescence (Figure 1, top). 

Accordingly, a model nitrone compound, (Z)-*N*-benzyl-α-phenylnitrone **1**, was synthesized [48] to compare fluorescent properties (Figure 1, bottom). The excitation spectrum of **1** in ethanol showed a maximum at 380 nm, similar to the Schiff-base crosslinked hydrogel. Emission maxima were observed at 415 and 435 nm (Figure 1, middle). The dual emission peaks of the nitrone were attributed to photochemical isomerization of the *Z* isomer to the oxaziradine intermediate via an excited singlet state.

The double emission peaks observed for **1** upon steady-state excitation are likely due to the nitrone’s rearrangement to an oxaziradine intermediate via the lowest singlet excited state. Various isomerization mechanisms and activation energy barriers have been published for aryl-substituted nitrones under photolysis conditions [49,50,51,52,53,54,55,56]. For example, photochemical-induced rearrangement of *N*,α-diphenyl-α-cyanonitrone to its corresponding oxaziradine occurred from the lowest singlet excited state when irradiated with 313 nm light. Oxaziradine formation was indicated by the decrease in nitrone absorbance at 322 nm and the emergence of a new band at 223 nm. Infrared spectra at 77 °K showed the disappearance of characteristic C=N and N→O nitrone stretching and the presence of oxaziradine ring vibrations [55]. Another group reported the photorearrangement of chiral aryl-substituted nitrones to optically active oxaziradines when excited at wavelengths greater than 300 nm [49]. Furthermore, photolysis studies of *N*,α-diphenylnitrone indicated a 20 nm hypsochromic absorbance band shift that was attributed to the singlet transition of the nitrone to its oxaziradine intermediate [52].

In the last decade, cell sorting and microfluidic technologies have enabled advancements on many fronts including the study of individual cells and the diagnosis of disease states [57,58,59,60,61,62,63,64,65,66,67]. Given both the favorable characteristics of hydrogels as artificial extracellular matrix scaffolds and the intrinsic fluorescence observed with these specific hydrogels, we evaluated this system to detect single cells via the abatement or loss of hydrogel fluorescence signal upon individual cell binding.

**Figure 2:**
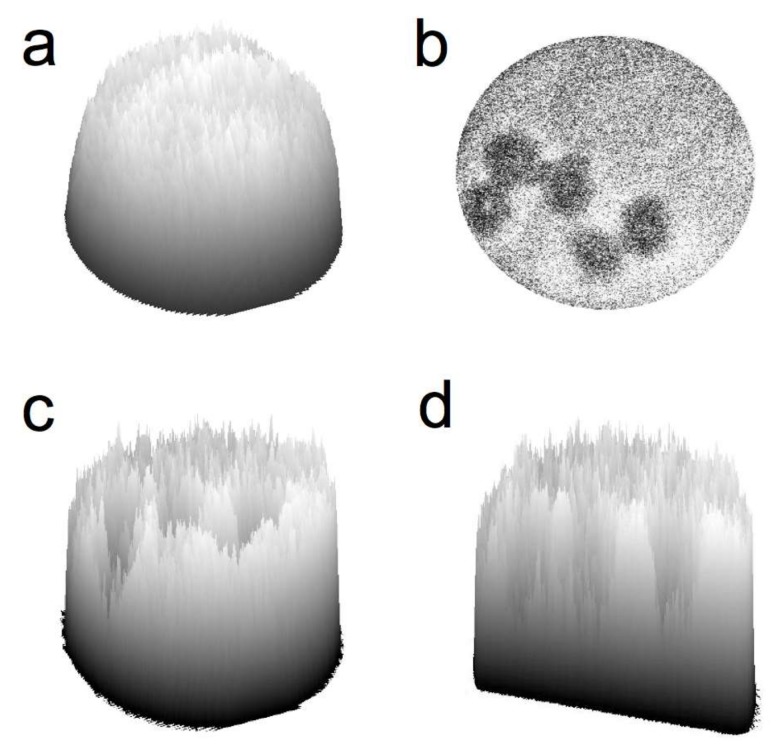
Scanning confocal microscopy images of BSA-treated hydrogels, 22 wt % 2:1 PEG-(CHO)_2_:PEG-(Lys)_2_. Emission was collected at 460 nm, subtracted from slide background, and normalized to 8-bits/pixel. (**a**) 3D surface plot of a hydrogel chamber in the absence of cell adhesion, (**b**) 2D depiction of cells masking hydrogel emission fluorescence, (**c**) 3D surface plot of the hydrogel shown in b, and (**d**) 3D cross-section of the hydrogel shown in **b**.

Specifically, printed hydrogel microarrays were treated with a bovine serum albumin (BSA) solution and excited by a 405 nm laser diode using a confocal microscope. The emission was monitored from 450 to 500 nm as transmitted through a beam splitter, and 3D photon surface plots of the hydrogel chambers were obtained (as shown in Figure 2a). Next, human A549 small cell lung carcinoma cells were incubated with the hydrogel microarrays and washed to remove unbound cells. As can be seen in the photon surface plots, the adhered cells significantly obstructed fluorescence throughout the entire hydrogel network with a hydrogel:cell signal-to-noise ratio of 3.4:1 (Figure 2b–d).

## 3. Materials and Methods 

### 3.1. General Information 

^1^H-NMR was recorded on a Varian INOVA at 400 MHz at ambient temperature. Chemical shifts are reported in parts per million as follows: chemical shift, multiplicity (s = singlet, d = doublet, t = triplet, q = quartet, m = multiplet, br = broad) and integration. Infrared spectra were recorded on a Nicolet Nexus 670 FT-IR ESP spectrophotometer. Analytical thin layer chromatography was performed using EMD 0.25 mm silica gel 60-F plates. Reverse phase chromatography was performed on a C8-bonded silica extraction column (United Chemical Technologies, Horsham, PA, USA). Sub-micro fluorimeter quartz cells were supplied by Starna Cells, Inc. (Atascadero, CA, USA). All solvents were purchased from Pharmco-AAPER or Fisher Scientific as highest purity grade or dispensed dry from a solvent purification system (CH_2_Cl_2_). Other synthetic reagents were obtained from commercial sources and used without further purification unless indicated. Benzaldehyde was distilled prior to use. NanoPure™ water (Barnstead International, Dubuque, IA, USA) was used for all microarray and cell processing procedures. 384-well plates were purchased from Genetix (Boston, MA, USA) and SuperAldehyde slides were purchased from TeleChem International (Sunnyvale, CA, USA). Cell culture and seeding materials were supplied by Fisher Scientific (Hampton, NH, USA) and Invitrogen (Carlsbad, CA, USA). Hydrogel microarrays were produced using an OmniGrid Accent™ Microarrayer (GeneMachines, San Carlos, CA, USA). Scanner fluorescence images were obtained on a GenePix 4000B microarray scanner and data analysis was done using GenePix 3.0 software (Axon Instruments, Union City, CA, USA). Fluorescence spectra were taken on a QuantaMaster™ Luminescence spectrofluorimeter and data was analyzed using FeliX32™ software (Photon Technology International, Birmingham, NJ, USA) and Microsoft Office^®^ Excel. Confocal fluorescence imaging of hydrogels was performed with an Olympus Fluoview™ 1000 scanning laser confocal microscope, controlled by Olympus FV10-ASW v1.61 software (Center Valley, PA, USA). Fluorescence emission data were acquired as the integrated photon intensity within a defined hydrogel region. Images were processed and analyzed with ImageJ [68].

### 3.2. Hydrogel Array Materials & Methods

Preparation of 2-iodoxybenzoic acid (IBX): Oxone (133.80 g, 0.218 mol) was added to a 1-L round bottom flask and suspended in 520 mL of distilled water. 2-iodobenzoic acid (30 g, 0.121 mol) was added in one portion and the reaction mixture heated to 70 °C and stirred for 3 h. The reaction was cooled to room temperature and then to 0 °C for 90 min to further precipitate the product. The resulting white solid was collected by filtration, washed with water (5 × 50 mL) and acetone (4 × 50 mL), and dried under vacuum to afford the pure product (27 g, 80% yield). The spectral data were in agreement with reported values [69].

**Figure 3:**
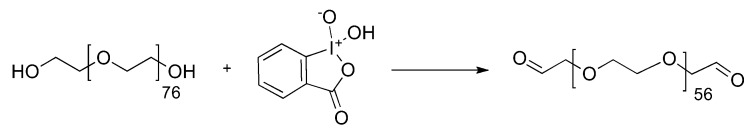
Synthesis scheme of the poly(ethylene glycol) dialdehdye (PEG-(CHO)_2_) macromonomer.

Preparation of poly(ethylene glycol) dialdehdye (PEG-(CHO)_2_) macromonomer (Figure 3): Poly(ethylene glycol) Mn = 3400 g/mol (3 g, 0.882 mmol) was dissolved in toluene (10 mL), concentrated under reduced pressure to a viscous residue, and dried under vacuum (2×). The residue was dissolved in a minimum of ethyl acetate (approx. 20 mL) and IBX (1.24 g, 4.4 mmol) was added in one portion. The reaction mixture was heated to 80 °C and stirred for 20 h. IBX was filtered over Celite and the filtrate was concentrated under reduced pressure to a thin residue. An additional filtration in ethyl acetate removed any remaining IBX. The product was concentrated under pressure and precipitated in cool ether for 2 h to afford crude PEG-(CHO)_2_ (0.8 g, 27% yield). The crude product was purified by reversed phase chromatography using a C8-bonded silica endcapped column (elution with 10–90% acetonitrile in water), and the product was lyophilized to afford pure PEG-(CHO)_2_. ^1^H-NMR (400 MHz, CDCl_3_): 3.62–3.72 (m, 340, CH_2_ of PEG); 4.14 (m, 2, CH_2_CHO); 9.71 (s, 1, CHO). 

Preparation of bis(2-amido-lysine)-poly(ethylene glycol) (PEG-(Lys)_2_) macromonomer: According to literature procedure [70].

Printing of hydrogel microarrays: The hydrogel solution (22 wt % 2:1 PEG-(CHO)_2_:PEG-(Lys)_2_ in HEPES buffer pH 7.4) was printed on aldehyde-coated glass slides, polymerized for 24 h at room temperature and blocked with a 1% *w/v* BSA solution in water as previously reported [33]. Fluorescence measurements were performed as described below. Attempts to observe the nitrone in the gel by IR were unsuccessful. 

A549 cell seeding conditions: Cells were cultured at 37 °C in a 5% CO_2_ atmosphere in Dulbecco’s modified Eagle medium (DMEM) supplemented with 10% fetal bovine serum and 10 μg/mL penicillin. Immediately prior to incubation with hydrogel microarrays, cells were detached from polystyrene culture flasks, centrifuged at 1000× *g* for 5 min, and re-suspended in the appropriate volume of phosphate buffered saline (PBS) to yield ~3 × 10^4^ cells/mL. The cell solution was slowly pipetted directly on top of the hydrogels in a 100 × 20 mm polystyrene culture dish and incubated for 20 min at 37 °C. Microarrays were then washed with PBS (10 mL) to remove unbound cells and tapped dry.

### 3.3. Fluorescence Image Analysis

GenePix 4000B microarray fluorescence scans: Following microarray printing, the macromonomers were allowed to polymerize for 24 h at room temperature. The crosslinked hydrogel chambers were excited with the microarray scanner and emission intensities were calculated as the mean intensity of the spot normalized to the background.

Steady-state fluorescence spectra: The hydrogel solution (14 wt % 2:1 PEG-(CHO)_2_:PEG(Lys)_2_) was contained in a 10 mm pathlength quartz cuvette under atmospheric conditions 24 h prior to fluorescence measurements. The samples were excited by a pulsed xenon lamp at a wavelength of 380 nm, and emission was monitored from 300–600 nm as the number of photon counts per second. Excitation and emission were controlled by a quarter-meter class, Czerny–Turner type monochromator with a standard 1200 line/mm ruled grating. Spectra data was corrected for solvent background, and counts per second were normalized to an arbitrary scale of 0–100.

Confocal fluorescence imaging: The hydrogel microarrays were printed and blocked with BSA solution as described above. For cell seeding hydrogel experiments, the microarray slides were further incubated with a solution of A549 human lung carcinoma cells for 20 min at 37 °C, washed with PBS, and tapped dry. Both blank and cell-seeded hydrogel arrays were imaged on a confocal scanning electron microscope with a 20× water-immersion objective lens. A 407 nm diode laser was used for excitation of the intrinsic hydrogel fluorescence, and detection was carried out between 450 and 500 nm at 20 μs/pixel resolution. Confocal emission spectra were generated using sequential emission imaging of the hydrogel at 5 nm bandpass between 440 and 600 nm. Maximum hydrogel emission, with minimal background interference, occurred at 460 nm.

Data were analyzed as the integrated pixel intensity (at 460 nm emission) within the area of a manually outlined hydrogel chamber, human cell, or background region. Fluorescence 2D images and 3D surface plots corresponding to the emission intensity of an outlined hydrogel chamber subtracted from slide background were constructed in ImageJ (Figure 2 in paper). Images were formatted to 8-bits per pixel (grayscale LUT) and contrast was enhanced. Signal-to-noise ratios were determined as the mean pixel intensity within a hydrogel area or cell area divided by the corresponding standard deviation.

### 3.4. Nitrone Model 

Preparation of *N*-benzylhydroxylamine [71]. A flame dried flask was charged with a stir bar and benzaldehyde oxime (1.00 g, 8.26 mmol) dissolved in methanol (9.03 mL, 0.223 mol) was added. Sodium cyanoborohydride (0.363 g, 5.78 mmol) was added and the pH was brought down to pH 4 with the addition of aqueous concentrated HCl. The reaction was stirred for 3 h. Solvent was concentrated under reduced pressure and crude product was diluted with 6 M potassium hydroxide and washed with brine. The organic layer was extracted with chloroform (3×) and dried over MgSO_4_. The product was concentrated and ^1^H-NMR spectroscopy showed sufficient purity to be used in the next reaction.

**Figure 4:**
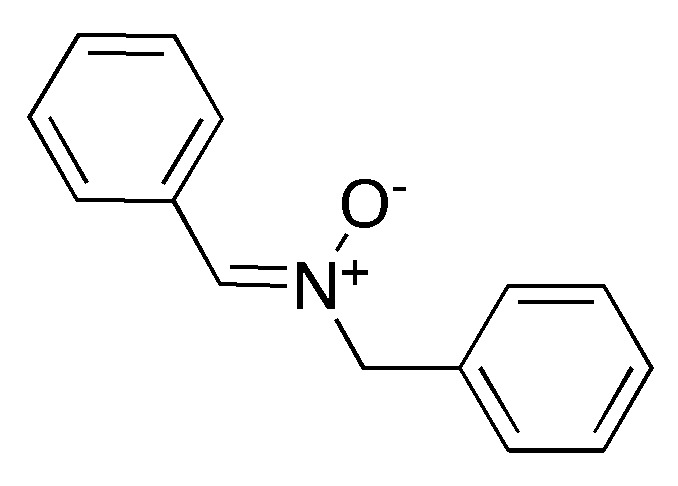
Chemical structure of nitrone, **1**.

Preparation of (Z)-*N*-benzyl-α-phenylnitrone (**1**; Figure 4): According to the procedure of Chang, et al. [48], a flame dried 5 mL round bottom flask was charged with a stir bar and flushed with nitrogen. To this flask was added anhydrous sodium sulfate (0.160 g, 1.125 mmol), *N*-benzylhydroxylamine (0.092 g, 0.75 mmol), benzaldehyde (0.111 g, 1.05 mmol), and dichloromethane (2.5 mL). The reaction was stirred under nitrogen at room temperature for 7 h. Sodium sulfate was filtered and the filtrate was concentrated under reduced pressure to give a light brown oil that solidified upon standing. The crude product was washed once with cold petroleum ether and recrystallized from hexane (2×) to afford 0.13 g (82% yield) of the *Z* isomer. The spectral data was in agreement with reported values [72].

Photochemical properties: Steady-state fluorescence spectra were taken of **1** (2.8 wt %, 0.13M) immediately following dissolution in either ethanol or dichloromethane. Fluorescence intensity was weaker and dual peak emission less pronounced in EtOH as compared to CH_2_Cl_2_ as result of the increased solvation of **1** due to H-bonding interactions. However, excitation and emission maxima were consistent between both solvents, and EtOH solvent fluorescence data was used for hydrogel fluorescence comparisons. The nitrone solution was contained in a 10 mm pathlength quartz cuvette and fluorescence measurements were taken under the same conditions as described for the hydrogel. Spectra data was corrected for background, and counts per second were normalized to an arbitrary scale of 0–100. 

## 4. Conclusions

In summary, fluorescence emission is observed from the hydrogels with a λ_max_ at 445 nm. The photochemical properties of these hydrogels are consistent with emission from a nitrone functionality formed via oxidation of the imine crosslinkages within the hydrogel. A new cell microarray assay is described, whereby the reduction or obstruction of fluorescence is monitored upon individual cell adherence to the hydrogel chamber. Typically, hydrogel parameters such as polymer wt % and crosslink density are altered for a specific application; now, fluorescence can be incorporated into such criteria by specific macromonomer selection. Continued experimentation with multifunctional dendritic macromolecules will lead to new polymer structures, crosslinked networks, and properties broadening the investigation of these unique macromolecules for basic and clinical applications.
